# Protocol for leading health services innovation: a hybrid type 2 mixed method implementation trial for developing and assessing a codesigned graduate certificate program in health innovation

**DOI:** 10.1186/s12909-024-06094-7

**Published:** 2024-11-05

**Authors:** Jennifer Kosiol, Mark Avery, Tracey Silvester, Michaela Smyth, Georgina Sanger, Jonathan Purdy, Stewart Alford, Joshua Byrnes, Andrea Bialocerkowski

**Affiliations:** 1 Griffith University, South Bank Campus, Brisbane, Australia; 2 Kaplan Business School Australia, Brisbane, Australia

**Keywords:** Health service innovation, Implementation science, Health workforce, Tertiary education codesign, Development protocol

## Abstract

**Background:**

A significant issue with innovative problem-solving in healthcare is an existing deficiency in continuing education for many healthcare professionals, which hinders the successful implementation of inventive solutions and progress in the field. Educators play a crucial role in guiding students to cultivate the knowledge and skills necessary to confront these challenges, including problem solving, collaboration, and the use of rapidly advancing technologies. It is vital to design educational programs that empower and motivate students to develop the proficiency and knowledge they need to be effective problem solvers, collaborators, and cultivators of innovative solutions. This project aims to assess the implementation and effectiveness of a codesigned postgraduate university program for a multidisciplinary health workforce.

**Methods:**

The Leading Health Services Innovation Project is a hybrid type 2 mixed method implementation trial of a codesigned Graduate Certificate in Health Services Innovation. In collaboration with a large tertiary and quaternary health service, we developed a codesign process to guide the project, with time quarantined to create space for two-way learning between health sector partners and healthcare academics. Qualitative interviews and quantitative surveys for primary users will evaluate the implementation strategies. The reach, effectiveness, adoption implementation, and maintenance (RE-AIM) framework will guide the evaluation and maintenance of the program.

**Results:**

Integrating a codesign strategy complemented by a well-structured implementation and evaluation protocol that is a combination of implementation science theoretical frameworks (Knowledge to Action, Evidence-Based Co-design, RE-AIM) may lead to translational competence as a potential outcome.

**Anticipated outcomes:**

The application, resourcing and commitment to codesigned tertiary-level learning and qualification will demonstrate the achievement of a contemporary and comprehensive postgraduate university degree program in health innovation management.

## Background

Globally, the cost of health care is increasing; however, despite increases in health expenditure, health service coverage is decreasing [1, 2]. In healthcare, ‘value’ is a system that optimizes efficiency while ensuring that the population can attain the highest level of healthcare for the expenditure invested [3]. Currently, health workforce leaders need to consider innovative service solutions to optimize value and efficiency. Innovative solutions are deliberate, coordinated and sustained solutions that enhance health outcomes, organisational efficiency, cost effectiveness and user experience [4]. The ever-evolving challenges, emerging health crises, and increasing demands on healthcare systems necessitate a proactive embrace of innovation. By exploring novel approaches, technologies, and strategies, the health workforce can not only enhance the quality of care but also streamline processes, improve resource utilization, and ultimately ensure the delivery of more effective and efficient healthcare services.

Preparing the current health workforce to address these healthcare challenges through education is critical for health sector innovation. It is essential that educators assist students in developing the knowledge and capabilities to meet these challenges, which include problem solving, collaboration, and the use of rapidly advancing technologies [5]. A critical concern involving innovative solutions to these challenges is that many current healthcare workers lack the training to develop or work with innovative solutions, thereby stalling successful implementation and advances in health. As such, it is imperative to design university programs that include education strategies that will enable and motivate students to develop the capability, knowledge, and skills they need to be effective problem solvers, collaborators, and cultivators of innovative solutions [6, 7].

It has been found that innovative education programs, those that include interdisciplinary approaches, real world problem solving, collaboration and peer learning with flexible blended learning options, can lead to increases in staff self-efficacy, confidence in problem definition, intervention design, knowledge translation, implementation, and research practices to support healthcare improvement and health service innovation [8].

### Codesigned curriculum development

Designing graduate certificate programs in universities traditionally involves a meticulous process facilitated by an academic faculty team that begins with a thorough needs assessment, an understanding of the target audience, and health sector requirements [9, 10]. For this study the target audience is health sector working professionals. The curriculum development phase involves defining clear learning outcomes and assessments aligned with health sector standards and selecting and organizing courses, delivery methods, and evaluation strategies [10]. However, navigating the intricacies of the healthcare system requires a comprehensive understanding of its multifaceted and interconnected components [10]. To address this issue, a codesign approach should be adopted in healthcare systems education to ensure that students comprehend how to tackle health care systems challenges effectively and efficiently using an academic framework.

Codesign curriculum development requires a collaborative relationship between health sector partners and universities, and the aim of this symbiotic relationship is to foster innovation and address contemporary challenges. This contributes to developing a skilled and adaptable workforce, which ultimately increases the effectiveness of patient care [11–13]. This collaborative partnership is predicated on the complementary strengths of each participant; universities contribute academic expertise, research capabilities, and freedom to explore cutting-edge technologies, while health sector partners provide real-world problems, market insights, and an emphasis on practical application. The cross-pollination of ideas inherent in codesign accelerates the pace of innovation and ensures the practical relevance of academic research by aligning it with health sector needs. The interplay between academia and health sector partners also facilitates the efficient use of resources, including sharing facilities and personnel, leading to cost-effective research initiatives [13].

In the context of the co-design process described, it is essential to clarify that the collaboration extends beyond the academic team and health sector partner administrators. Alumni students both from the industry partner and the university, are also actively involved in the co-design process, ensuring that their perspectives and needs are integrated into the development of the curriculum. By including a diverse range of student voices, the co-design process aims to create a curriculum that is not only relevant and practical but also tailored to meet the evolving needs of the healthcare workforce.

However, the codesign process must contend with the concept of tribalism, which refers to the tendence of individuals or groups within a particular community, such as professional sectors, academic institutions, or healthcare system, to prioritize their own perspectives, beliefs or methodologies over others [14–16]. In the context of this study, tribalism can manifest as a strong identification with a particular group’s approach to solving problems, potentially leading to resistance against new ideas or innovations proposed by external parties.

Addressing tribalism is crucial for effective stakeholder engagement. The need for a co-designed Graduate Certificate suggests a deliberate attempt to break down the silos (tribes) that exist within the healthcare workforce and academic sectors [14, 16]. Tribalism might lead to resistance from certain stakeholder groups who prefer maintaining control over the curriculum or methodologies they are accustomed to rather than adopting new, collaborative frameworks [14, 16]. Furthermore, as the program aims to integrate diverse perspectives from various health sectors, tribalism could hinder collaboration across disciplines. Individuals entrenched in their specific "tribal" practices might resist interdisciplinary approaches, which are essential for fostering innovative solutions in healthcare.

To overcome these challenges, it is vital to foster inclusive dialogue, transparency, and a shared vision across the different "tribes" or professional groups involved. This approach encourages broader buy-in and collaboration for the co-designed program, ensuring that all relevant stakeholders feel their expertise is valued in the program's design and implementation. By addressing tribalism within the co-design framework, we can enhance the likelihood of successful outcomes and create a more cohesive and innovative educational environment.

Additionally, a codesigned approach has broader implications for talent development and technology skills transfer. It provides students with invaluable opportunities to engage in real-world projects, enhancing their practical skills and employability. It also allows crucial and early engagement to identify potential challenges, thereby minimizing risks within the curriculum and increasing the likelihood of successful outcomes. This study protocol includes an outline of the procedure through which an innovative, collaborative program that considers health services innovation will be codesigned with health sector partners and how it will be implemented and evaluated.

### Development of translational competence

Considerable attention has been devoted to exploring the characteristics of the translational gaps between healthcare science and service delivery [17]. The nature of the healthcare science-to-service gap underscores a critical disconnection between scientific advancements in healthcare and their effective translation into practical, patient-centered services. This gap is characterized by a lag in incorporating cutting-edge research findings and technological innovations into routine clinical practices [17]. While the scientific community continues to make significant strides in understanding diseases, developing novel treatments, and advancing diagnostic technologies, the integration of these initiatives into everyday healthcare delivery remains a significant challenge [17]. Factors contributing to this gap include regulatory hurdles, limited resources for technology implementation, and a slow diffusion of evidence-based practices. Consequently, there is a disparity between the potential of healthcare science and actual service delivery. Addressing this gap necessitates concerted efforts to facilitate the effective translation of research outcomes into clinical application, fostering a more responsible and efficient healthcare system that maximizes the positive impact of scientific advancements. To do this, a health workforce that is better equipped to adapt to rapidly evolving medical and technological advancements is needed.

A healthcare workforce focused on and supported in innovation is crucial for overcoming emerging health threats and global pandemics. The ability to respond quickly to new and unforeseen challenges requires a workforce that can think creatively, implement novel strategies, and leverage technology to address public health crises effectively. Innovation in healthcare is also instrumental in improving patient experiences, enhancing the quality of care, and increasing access to healthcare services through the development of telemedicine, digital health solutions, and other transformative technologies [18].

Fostering an innovative healthcare workforce also contributes to attracting and retaining top talent. Healthcare professionals who are encouraged to think creatively and contribute to advancements in their field are more likely to be engaged, motivated, and satisfied in their roles. This, in turn, leads to a more dynamic and resilient healthcare system that can better meet the diverse and evolving needs of patients and communities and ensure the best possible care for patients.

### The merging of codesign protocols for translation with the principles of implementation science

Implementation science is a multidisciplinary field dedicated to the study of systematic processes and strategies to integrate evidence-based interventions into real-world settings to improve health outcomes [19]. It employs a systematic approach with a focus on the complex factors that influence the successful incorporation of innovation and considers variables such as organizational structures, policy frameworks, and individual behaviors [20]. By identifying facilitators and developing and evaluating tailored implementation strategies, the aim of this field of science is to optimize the adoption, implementation, and sustainability of evidence-based interventions, ultimately fostering more efficient and equitable healthcare delivery.

Researchers have generated several implementation science theories, models and frameworks to understand and improve the adoption of evidence-based practices. Findings from an international survey of implementation scientists identified more than 100 theories, models and frameworks [21]. The most frequently used implementation science frameworks included the Consolidated Framework for Implementation Research (CFIR); the Theoretical Domains Framework (TDF); Promoting Action on Research Implementation in Health Services (PARIHS); the Diffusion of Innovations; Reach, Effectiveness, Adoption, Implementation, and Maintenance (RE-AIM) framework; the Quality Implementation Framework; and the Interactive Systems Framework [21]. Selecting appropriate theories/frameworks to guide practice is crucial because ineffective application can lead to the development of inappropriate implementation strategies. This can affect the translation of knowledge into practice, thereby restricting the impact and improvements in the healthcare sector [21, 22]. To address this issue, Nilsen [23] systematically classified emerging implementation science theories as having three distinct aims: 1) those guiding the process of transforming research into practice; 2) those that explain the factors that influence implementation outcomes; and 3) those that focus on evaluating implementation.

Many studies have used implementation science theories, models and frameworks to guide the codesign of public health and health ‘behavior change’ education programs for end users of a service; however, the end user in these studies is often the patient [24]. The use of implementation science theories for codesign within postgraduate training programs for the health workforce is less prominent [25–28].

**Fig. 1:**
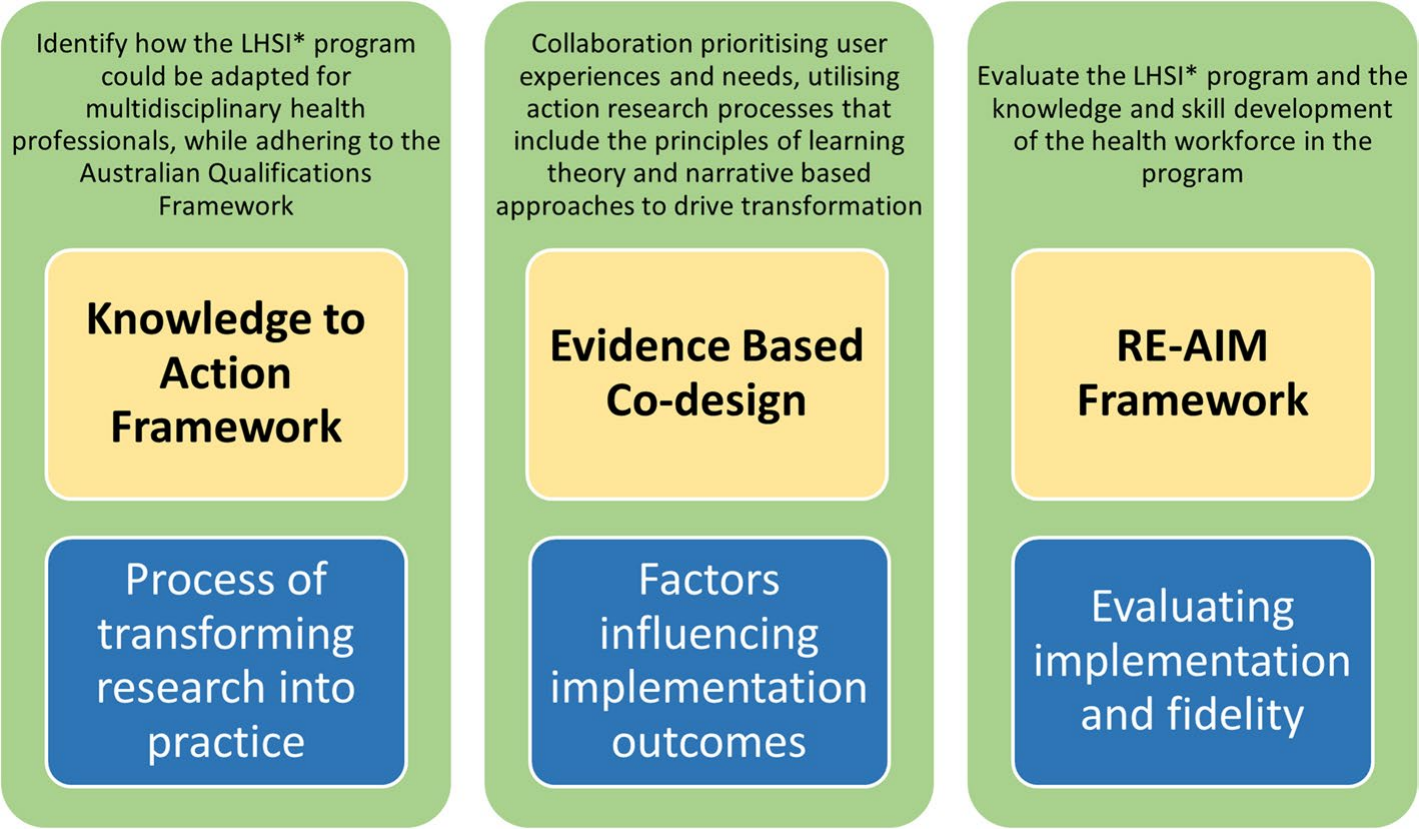
The integration of translational protocols with implementation science theories to design a postgraduate program in health services innovation (developed from findings in Nielsen [23]). *LHSI – Leading Health Services Innovation

Prior to developing the protocol for this project, the authors used Nielsen's [23] guide to identify an implementation theory, model or framework that could be tailored to address each specified aim. of the project. The Leading Health Services Innovation (LHSI) Project leaders adopted (a) the Knowledge-To-Action (KTA) framework to guide the process for effective transfer strategies for knowledge translation; (b) Theoretical Domains Framework (TDF) to identify the factors impacting the implementation; and (c) the RE-AIM framework to evaluate the implementation (see Fig. 1).

## Methods and analysis

### Theoretical framework

The KTA framework is a model designed to facilitate the translation of research findings into practical applications, particularly in the context of health-related innovation [23, 29]. Comprising knowledge creation and action cycle components, the framework guides a systematic approach, emphasizing the adaptation of interventions to specific contexts and incorporating stakeholder involvement (e.g., education modules and skill development for practitioners). It promotes evidence-based practices and iterative processes, allowing for continuous improvement as interventions are implemented and evaluated. By providing a structured pathway from knowledge synthesis to practical implementation and feedback, the KTA framework is a valuable tool for the healthcare sector, ensuring that innovations are grounded in research, contextually relevant, and can lead to a positive impact on health outcomes. For example, as part of the knowledge creation phase, the project team methodically examined both health innovation and learning and teaching strategies to identify how the graduate certificate program could be adapted to include new, flexible and authentic learnings for multidisciplinary health professionals while adhering to the standards for qualifications in education in the Australian Qualifications Framework [30].

Subsequently, the academics adjusted their acquired knowledge to the local context by explicitly evaluating the challenges and enablers affecting the application of curriculum design as part of the action cycle of the KTA framework. For example, newly developed curricula, assessments, and educational tools were codesigned and directed by feedback from alumni, HealthInnTrans (the university’s established health sector advisory network), university learning and teaching experts, and health service management discipline academics. The process of feedback was iterative and initially linear, but we found that we needed the flexibility of returning to groups with specific questions. This provided the necessary flexibility to adapt design knowledge to the specific context.

Experience-based codesign (EBCD) is a methodology that actively involves end-users, including consumers and healthcare providers, to design and improve healthcare services [31]. This collaborative methodology prioritizes user experiences and needs by utilizing a participatory action research process that includes the principles of learning theory and narrative-based approaches to drive transformation. The user-centered approach of EBCD ensures that innovations directly address the real challenges faced by individuals in the healthcare system, enhancing the quality of care and increasing the likelihood of successful adoption. The EBCD process typically includes understanding personal experiences, identifying critical touchpoints, engaging in codesign workshops, and reviewing what has been developed. The codesign process allows all stakeholders to make meaningful contributions. For example, in this project, one crucial touchpoint was including authentic assessments that were not predominantly essay writing. The academic team developed assessment prototypes to align with the Australian Qualifications Framework, and health sector partners provided feedback on these prototype assessment tasks, which assisted them in understanding university requirements.

The RE-AIM framework, encompassing Reach, Effectiveness, Adoption, Implementation, and Maintenance, is a comprehensive tool used to assess the impact and sustainability of the implementation process [32, 33]. Particularly beneficial for codesigned education programs on health innovation, RE-AIM is a holistic approach that considers diverse dimensions, including inclusivity in reach, effectiveness in achieving goals, adoption by relevant entities, implementation fidelity, and long-term maintenance [32, 34]. This framework aligns with the principles of codesign, ensuring that interventions are not only effective but also accessible, acceptable, and sustainable over time. In this study, RE-AIM was used to evaluate the graduate certificate program and the knowledge and skill development of the students in the program.

### Study design

Leading Health Services Innovation (LHSI) is a bespoke program designed collaboratively with health sector partners to support the health workforce in innovating, transforming and leading improvements in the healthcare sector. The LHSI program includes a codesigned Graduate Certificate in Health Services Innovation, micro credentialled stackable short courses, roundtables, and conferences. The Graduate Certificate in Health Services Innovation comprises four codesigned courses (syllabus subjects) that combine elements of leadership, facilitating innovation, and achieving successful implementation of new ways of working. The Graduate Certificate will be delivered part-time over two years and is offered in a blended learning mode, combining online, face-to-face and self-directed learning modes.

The aim of this project is to assess the implementation and effectiveness of the codesigned Graduate Certificate in Health Services Innovation for a multidisciplinary health workforce. The program is designed to support the health workforce in innovating, transforming, and leading improvements within the health sector.

In developing this graduate certificate program, we aimed to acknowledge the translational gap between healthcare science and service delivery as a crucial step in codesign. Specifically, tiered codesign protocols were designed to guide the project. This included acknowledging the need to allocate sufficient time and space for feedback from all stakeholders to support the development of a comprehensive understanding of different perspectives related to needs, evidence-based practice, and practical barriers in service delivery. For example, through face-to-face informal meetings, health sector partners provided competence training on practical challenges to the research team. Reciprocally, the academic team provided competence training to health sector partners on obligations such as the standards required for Australian qualifications at the graduate certificate level.

Additionally, the academic team recognized that developing trust, confidence, and a sense of ease was dependent on the quality of the relationships and the mutual connection that comes from a shared vision of developing an innovative health workforce. Robust connections developed because of frequent, sometimes daily, discussions between academics and key health sector personnel, reinforcing the strength of the relationship despite being situated in separate locations. Overall, the codesign process needed to be comprehensive, including attention to input from past alumni, local health sector experts, academic health service experts, learning and teaching curriculum development specialists, and a wider healthcare sector network.

#### Project aims

**Fig. 2:**
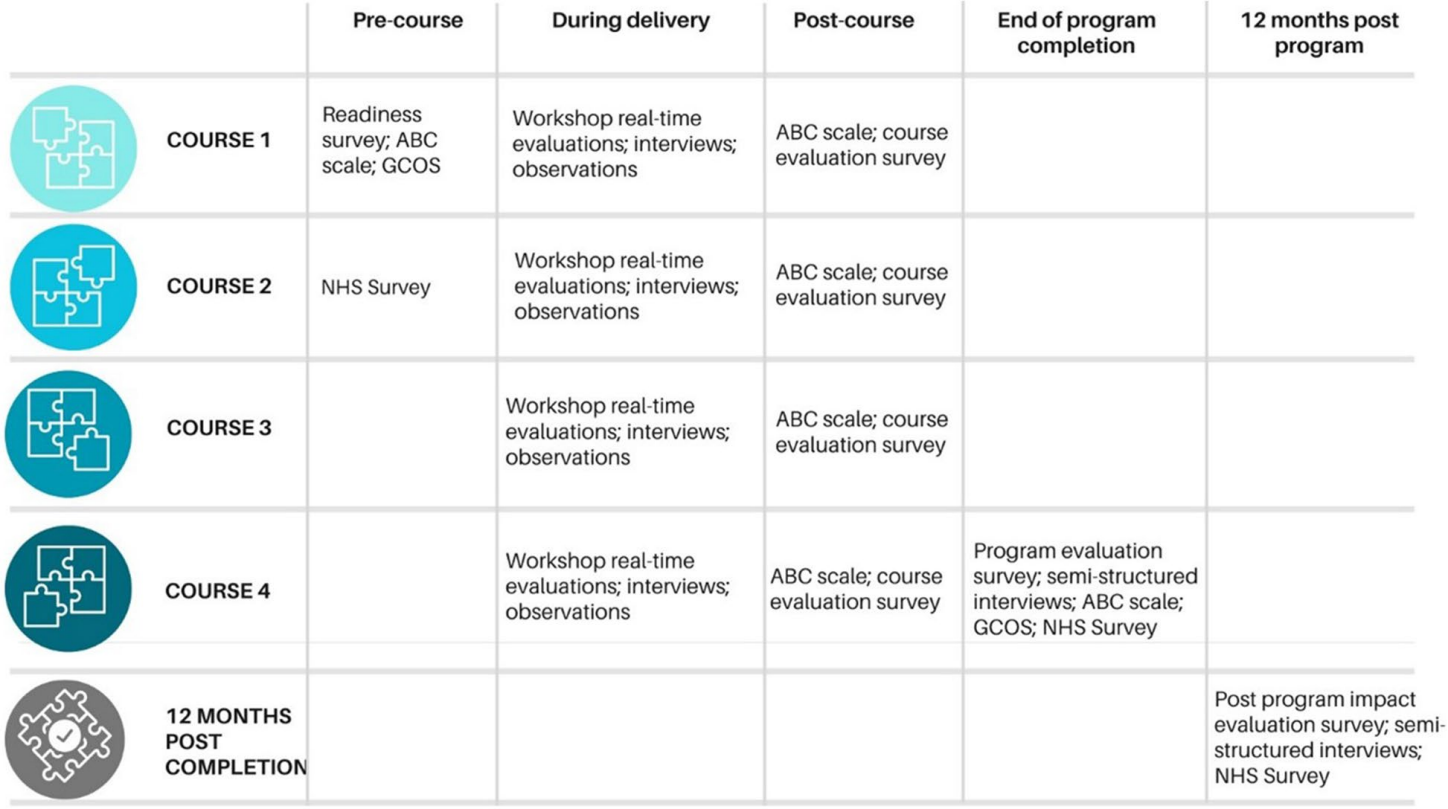
Evaluation phases of the LHSI program

This project will use- KTA theory and EBCD to:

1. Co-design a graduate certificate program that incorporates healthcare innovation strategies and principles to provide the health workforce with knowledge, skills and ability to implement accessible, acceptable, and sustainable healthcare improvements. Success will be measured by the completion of the program syllabus, including four co-designed courses and feedback from stakeholders involved in the co-design process. See Fig. 2 for the evaluation phase.
2. Design learning and teaching strategies to develop translatable knowledge and skill development. Effectiveness will be assessed through student evaluations, see Fig. 2.
3. Design authentic assessment that builds on previous knowledge and skills and aligns to Australian Qualifications Framework level 8. The success of the assessments will be evaluated based on student performance metrics.

The evaluation of the project will include the following principles of the RE-AIM framework to:

1. Deliver the graduate certificate courses using evidence-based knowledge and skill development strategies through workshop and online delivery.
2. Explore how co-designed innovation programs can motivate students to achieve competence and confidence in innovation implementation within their health service.
3. Evaluate the implementation and efficacy of the Leading Health Services Innovation program throughout each phase of the project.

### The study setting and population

Our partner is one of the largest public hospital and health services (HHS) in the Southern Hemisphere, comprising the full scope of health services, including quaternary and tertiary hospitals, community-based health services and aged care. It is a statutory body governed by a board of directors and is a recognized leader in the provision of world-class healthcare, with expertise in numerous specialties that provides the leverage to expand and drive healthcare forward through research, technologies, and partnership opportunities.

The Academic Team comprises experts from the health services management and health economics disciplines.

Research priorities include knowledge translation and health services innovation through the delivery of an innovative program to connect clinical and business decision-making. The health workforce from this health service and from other Queensland Health Hospital and Health Services have the opportunity to undertake the newly codesigned Graduate Certificate in Health Services Innovation.

### Study design

A mixed methods hybrid type 2 [35] clinical effectiveness and implementation trial was adopted for this study. This method is designed for dual testing of clinical intervention (workforce education) and implementation strategy (academic detailing). Direct blending of clinical effectiveness and implementation research aims to improve the speed of knowledge creation and support rapid translation. This hybrid design is motivated by the acknowledgment that traditional effectiveness studies tend to produce effectiveness estimates that are notably different from, and often worse than, the estimates obtained from efficacy studies [35]. The reason for this difference is attributed to the fact that effectiveness studies are frequently conducted under challenging, often “worst case”, conditions. These conditions include limited or no support from the research team during delivery and implementation, a lack of clear understanding about fidelity barriers, and a failure to make efforts to overcome these obstacles. In essence, the hybrid approach addresses these shortcomings and improves the accuracy of effectiveness estimates by incorporating elements from both efficacy and effectiveness studies. In the current hybrid type 2 study, the implementation intervention strategy (health workforce-led improvements) will be evaluated alongside and in support of the codesigned graduate certificate program. This creates a “medium case”, or “pragmatic”, set of delivery and implementation conditions that lie between the best and worst cases, which acknowledges that in real-world applications, studies often encounter a mix of challenges and support.

### Sequential phases of data collection and analysis

This study design will involve multiple phases where qualitative and quantitative data are collected and analysed in different phases, allowing for the systematic integration of findings overtime. For this study, it will provide flexibility to adjust based on early findings, making it a good fit for a trial that balances clinical effectiveness and implementation outcomes [36]. In the final integration phase, findings from both clinical intervention and implementation strategy assessments will be synthesised to inform refinements to both components. This process will account for the “medium case” conditions under which the intervention was delivered and implemented, ensuring that real-world challenges and supports are considered.

This approach facilitates iterative testing and continuous refinement based on real-time data, aligning with the hybrid trial objective of accelerating knowledge translation and improving the accuracy of effectiveness estimates in pragmatic settings [36].

### Sample size

Primary users of the program are the Hospital and Health Service (HHS) health workforce, defined as practitioners and administrators who are trained by the project team (i.e., administrative support officers, nurses, medical practitioners, allied health) and who are enrolled in the graduate certificate. The two cohorts will commence the program one year apart. Each cohort will consist of staff selected by the HHS with up to forty (40) places per cohort. Cohort 1 commenced July 2023 in the LHSI program, which consists of the Graduate Certificate of Health Services Innovation, three roundtables, and a yearly conference. Cohort 2 commences in July 2024.

### Ethics

Ethical approval for this study was obtained from Griffith University (GU Ref No: 2023/566) and the Townsville Hospital Human Research Ethics Committee (HREC/2023/QTHS/100088). This research is considered negligible risk; however, the following key ethical issues have been identified: informed consent and power dynamics; and confidentiality [37, 38]. Informed consent and power dynamic is provided by the participants in the study where they will sign the purposefully designed consent form prior to the commencement of the interviews. The consent form will be contextualized to prevent participants feeling threatened by the format of a standard consent form. In addition, a participant information letter will be provided, outlining the purpose of the study, financial implications, confidentiality, consent implications and support network. Confidentiality is established by the researchers in order to protect the privacy of the individual participant and participating organisations. By using aggregated results in published work, and de-identification of organisations and participants, confidentiality will be maintained. Informed consent will be obtained from all participants prior to any involvement in the study. Consent for online anonymous surveys will be implied by the respondent completing the survey.

### Codesign stakeholder group protocol

Program development procedures, materials and assessments will be discussed at regular meetings with a stakeholder group that represents alumni of a previous graduate certificate program undertaken by the HHS, HHS executive and research practitioners, HHS experts and educators, healthcare practitioners, health services and health economics academics, learning and teaching curriculum development experts and the HealthInnTrans network (health sector experts).

### Data collection and analysis

#### Qualitative methods

According to the stage of the project, qualitative interviews will be conducted to identify a) workforce strengths and needs, b) current barriers to healthcare improvements, c) the value of the educational program and each course, d) the efficacy of knowledge translation, and d) satisfaction with the program. A thematic analysis of the interview transcripts and observation narratives will be used for its iterative process of making sense of the data [39]. Themes will be derived from the coded data, this process will be undertaken by two researchers independently. Information will be gathered regarding the intervention’s content, structure, operational methods and any potential changes over time. In addition, the experiences of healthcare professionals, along with facilitators and barriers will be explored. These analyses will provide insights to the projects aims and will assist with quality improvement of the program delivery, educational modules, and fidelity beyond the research project.

#### Quantitative methods

The National Health Service (NHS) Creating a Culture for Innovation tool [40] will be administered at three timepoints: at the start of the program, at the conclusion of the four courses of the Graduate Certificate and at 12 months post completion for each cohort.

In addition, an adapted version of the Academic Behavioral Confidence (ABC) Scale and the General Causality Orientations Scale (GCOS) [41] will be used at the beginning and end of each of the Graduate Certificate programs for both cohorts 1 and 2 (30–40 participants per cohort) (Fig. 2). The sample size will be restricted by the number of individuals enrolled in the Graduate Certificate. The ABC scale measures constructs of self-concept and self-efficacy and is a psychometric means of assessing the confidence that students have in their own anticipated research skills and study behaviors in relation to their degree program. The understanding that students have of their research skills can be important for making sense of their expectations of teaching, learning and assessment [42, 43]. The adapted ABC scale consists of 10 items that will allow for responses on a 5-point Likert scale. Data analysis will involve descriptive statistics to summarize the data collected from the NHS Creating a Culture for Innovation tool, the Academic Behavioral Confidence (ABC) Scale, and the General Causality Orientations Scale (GCOS). Changes over time will be examined by comparing results across the three timepoints for the NHS tool and between the beginning and end of the Graduate Certificate programs for the ABC and GCOS scales. Paired t-tests or repeated measures ANOVA will be conducted to assess any statistically significant differences in motivation and confidence levels across the timepoints [44]. Additionally, correlation analyses may be performed to explore relationships between participants' self-efficacy, confidence, and motivation in relation to research and innovation skills. The data collected from these instruments will be used to assess the motivation and confidence levels of the participants in relation to research skills, specifically innovation and implementation, skills and knowledge.

### Implementing the codesign process

**Fig. 3:**
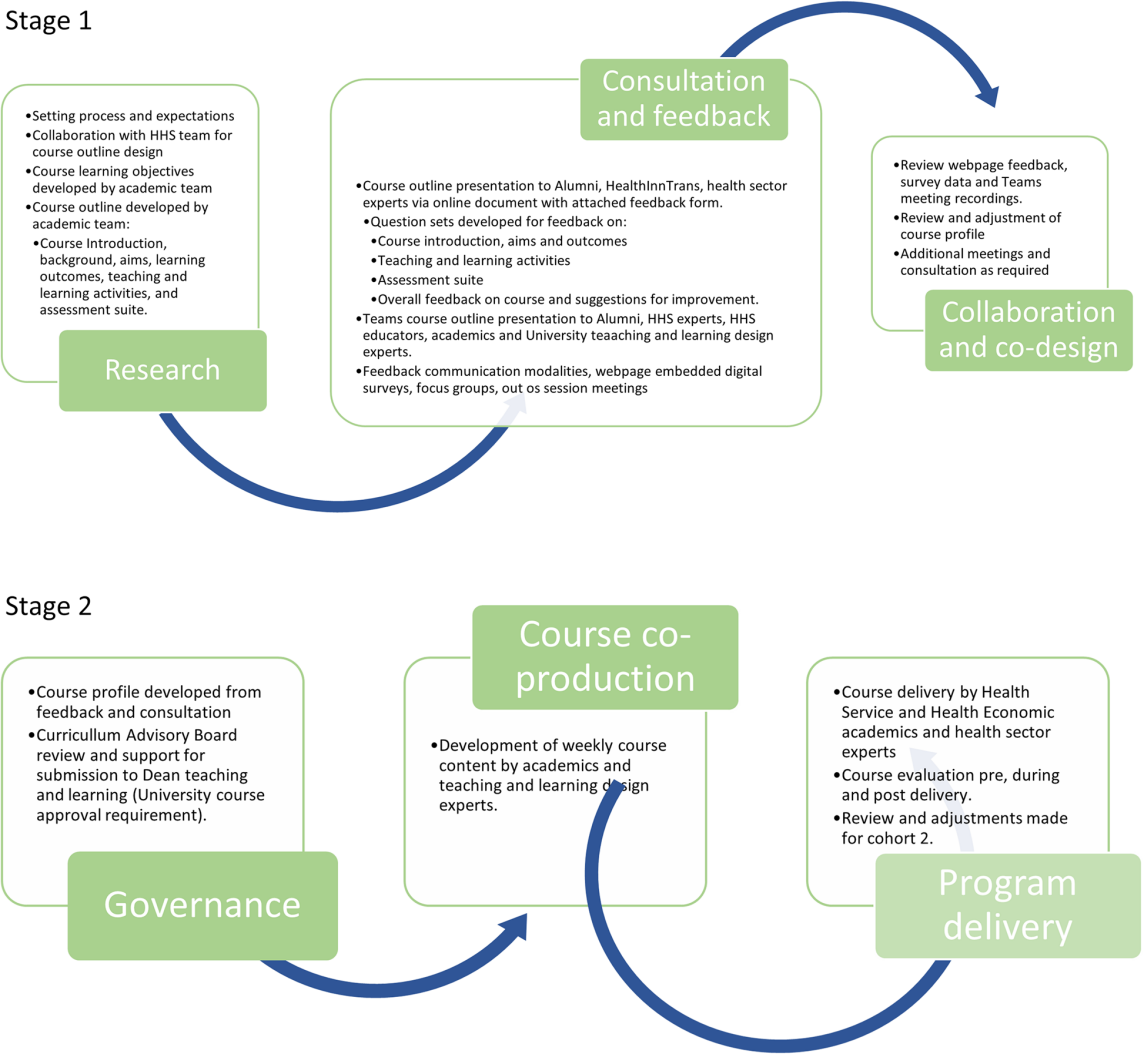
Spectrum of codesign approach

Codesign of the graduate certificate program began early in the project, with the healthcare organization providing an outline of its aims and objectives for each of the four courses in the program. The university then provided an outline of learning outcomes and scaffolded skill development for each of the courses to the health sector partner to form a basis for starting the codesign process. Once the outlines were agreed upon, course design commenced by codesigning a course profile (subject information). The course profile outlines the course aims, learning outcomes, knowledge and skill development, and assessment criteria. All of these components are codesigned to ensure that the program is responsive to health sector needs. The stakeholder codesign spectrum, shown in Fig. 3, is designed to include more than just consultation and feedback and includes active stakeholder involvement in identifying issues and potential solutions.

The codesign spectrum will be implemented at different stages for the four courses in the graduate certificate. Stage 1 of the codesign process involves the development of a course outline and profile by the academic team. Health sector partner approval is then required to proceed to key stakeholder presentations (alumni, HHS experts, HHS educators, academics and university learning and teaching design experts). A review of the feedback and adjustments are then made to the course outline before proceeding. Stage 2 of the codesign process involves the presentation of the adjusted course profile to the Curriculum Advisory Board (CAB) (University and HHS executives governing the LHSI program). Reviews and further adjustments are made, and the manuscript is resubmitted to the CAB for final approval and support for submission to the Dean Learning and Teaching for university approval. Following this, the course will be developed within an online platform. A local project manager coordinated the codesign process through stage 1 and stage 2.

### Evaluating the hybrid type 2 implementation trial

To allow for dual testing of clinical (workforce education) and implementation (academic detailing) interventions and strategies, the project will follow the RE-AIM framework to evaluate the LHSI program. A detailed description of the assessments of the RE-AIM domains is provided in Table 1.

**Table 1:** Hybrid type 2 evaluation framework

Reach	LHSI numbers including student participants in the graduate certificate of health service innovation; health sector supervisors, line managers and executive who participate in mentoring, supervision and sponsoring of students; practitioners and key stakeholders who participate in the LHSI program including workshop delivery, roundtables, conferences; and academic practitioners involved in development and delivery
Effectiveness	Students • Knowledge and skill development in health service innovation • Student confidence and motivation development throughout the program • Student confidence and knowledge development for assessment • Student workshop participation, knowledge and skill development • Quantitative information will be collected regarding: ∘ Workshop effectiveness ∘ Confidence and motivation ∘ Satisfaction of content delivery and teaching and learning activities • Qualitative information will be collected regarding: ∘ Satisfaction with course content and delivery ∘ Changes in teaching, learning and assessment reported by students ∘ Innovation and translation knowledge and skill development reported by students Key stakeholders • Codesign process evaluation reported by key stakeholders • Program evaluation **•** Project innovation development evaluation
Adoption	The number of innovation projects implemented throughout the health service Adoption of translational learning within the organization
Implementation	The delivery of the LHSI program including two cohorts through the graduate certificate in health service innovation. The extent of the intended delivery and the number of iterations required through the codesign process. The teaching and learning design strategies and educational tools needed. The facilitators and barriers to implementation of the codesign, curriculum development, cohort delivery, as an indication of the degree of adherence to the prescribed program and to understand the effectiveness and reliability of the program and interventions implemented as intended and whether deviations impact outcomes
Maintenance	The number of innovative strategies implemented into the workplace and positive innovative strategies developed and adapted

### Project timeline

**Fig. 4:**
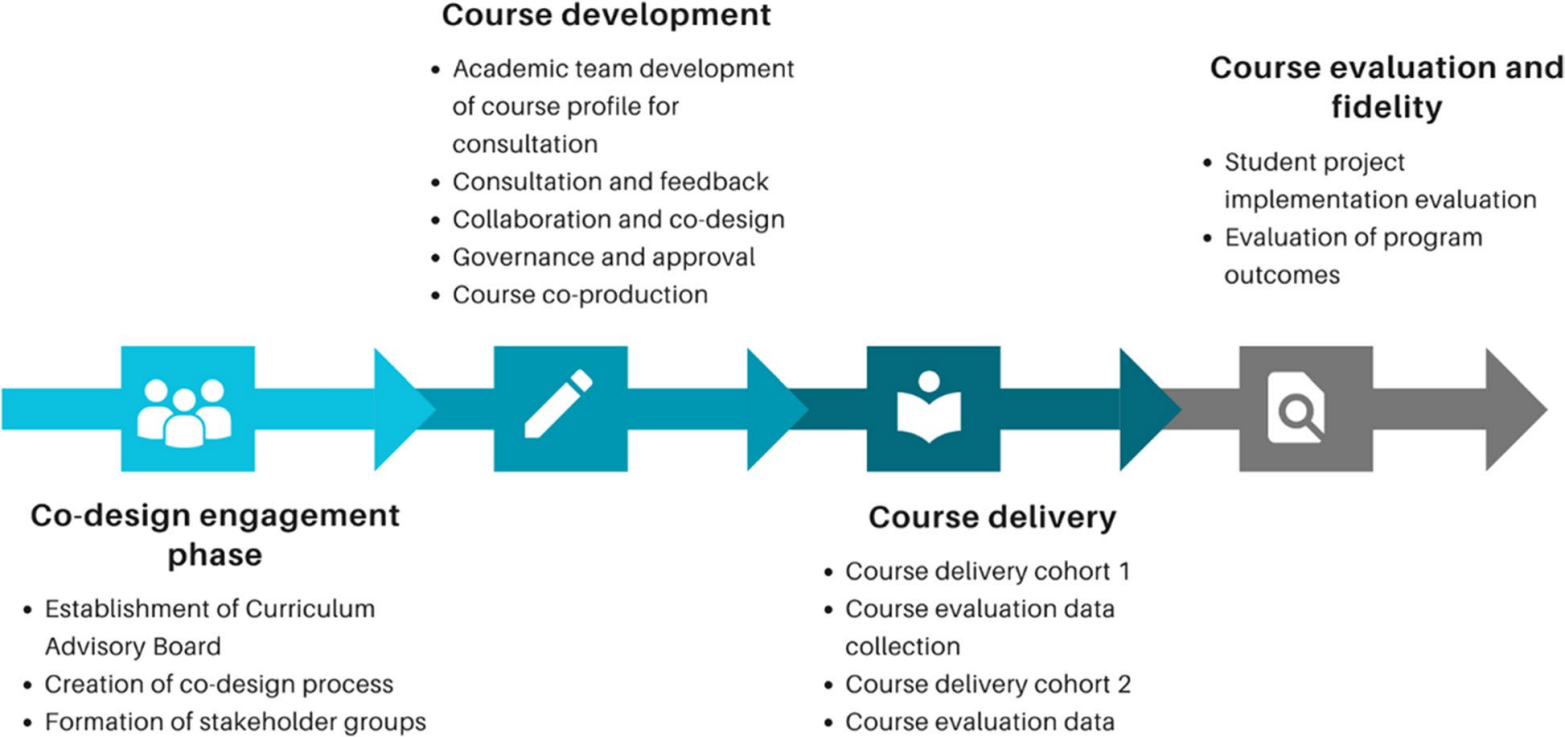
LHSI codesign project approach

The project began in January 2023 and will continue through July 2027. The first two months involved establishing the codesign process and associated engagement phase of the project. During this period, a Curriculum Advisory Board was established to oversee governance of the project. The next phase involves course codesign, development, and delivery. Ongoing codesign, development, implementation, and evaluation will continue throughout the project. The proposed codesign approach is outlined in Fig. 4.

## Discussion

Ongoing scholarly debates persist on how to define, apply, or distinguish between evidence-based and evidence-informed practice [45–50]. However, it is widely acknowledged that the mere presence of evidence does not guarantee its automatic application in practical settings [46, 48]. This study protocol includes a theoretical framework to explore and explain the mechanisms through which this implementation study is expected to influence the intended outcomes and impact of this study.

Several key aspects of the codesign strategy will play a pivotal role in shaping the program, including the depth of diversity in stakeholder contributions, the relevance of the program to the health sector, the incorporation of an interdisciplinary approach, the recognition of the time-consuming nature of codesign as an investment for cost‒benefit, and the commitment to a rigorous quality assurance process.

We developed and implemented a robust evaluation protocol that incorporates theoretical frameworks of Knowledge to Action, Evidence-Based Co-design, and RE-AIM. This amalgamation of theoretical perspectives provides a synergistic approach to implementation studies, offering a comprehensive understanding of both the processes and the anticipated outcomes. Knowledge to Action provides a structured pathway for translating evidence into practice, ensuring that the program is grounded in the latest research and health sector insights. Evidence-based codesign integrates the perspectives of end-users, fostering a user-centered approach that enhances the relevance and usability of the program. The RE-AIM framework, with its emphasis on reach, effectiveness, adoption, implementation, and maintenance, guides the evaluation process, offering a holistic lens to assess the program's impact at different stages. This integrated approach not only strengthens the theoretical underpinnings of this implementation study but also maximizes the potential for meaningful and sustainable outcomes in the field of health service innovation.

Codesign is a time-consuming process that requires extensive collaboration, feedback loops, and iterations. However, we view this as a deliberate and necessary investment in the overall impact and outcomes of the program. Our investment in codesign allows for thorough exploration of ideas, refinement of content, and validation of approaches.

### Anticipated outcomes

Current research and discussion surrounding evidence-based and evidence-informed practice highlight the complexity of translating research into practical education opportunities and healthcare settings. While acknowledging that evidence alone does not ensure automatic application, this study protocol proposes a useful theoretical framework for an implementation study, focusing on the development of a codesigned Graduate Certificate degree program in Health Services Innovation. The chosen codesign approach prioritizes stakeholder involvement, diversity, interdisciplinary collaboration, and rigorous quality assurance. Despite the time commitments needed, commitment and investment in codesign are crucial for producing a relevant, sustainable program that is demonstrating impact. The use of Knowledge to Action, Evidence-Based Co-design, and RE-AIM frameworks enhances this study's theoretical underpinnings. This enables a structured pathway for translating evidence, a user- and content-focused approach, and a comprehensive evaluation process. The codesign strategy aims to maximize the potential for meaningful and sustainable learning outcomes for leaders and managers in health service innovation.

## Data Availability

Data sharing is not applicable to this article, as no datasets were generated or analyzed during the current study.

## Abbreviations

REAIM framework: Reach Effectiveness, Adoption, Implementation and Maintenance Framework
WHO: World Health Organization
CFIR: Consolidate Framework for Implementation Research
TDF: Theoretical Domain Framework
PARIHS: Promoting Action on Research Implementation in Health Services
KTA: Knowledge to action
LHSI: Leading health services innovation
EBCD: Experience-based codesign
HHS: Hospital and health service
NHS: National Health Service
ABC: Academic Behavioral Confidence Scale
GCOS: General Causality Orientation Scale
CAB: Curriculum Advisory Board

## Data availability

No datasets were generated or analysed during the current study.
